# Laparoscopic mesh repair and Toupet fundoplication for parahiatal hernia complicated by sliding hiatal hernia: A case report

**DOI:** 10.1016/j.ijscr.2022.107664

**Published:** 2022-09-14

**Authors:** Risa Muramatsu, Takayuki Nobuoka, Tatsuya Ito, Tadashi Ogawa, Takahiro Korai, Ichiro Takemasa

**Affiliations:** Department of Surgery, Surgical Oncology and Science, Sapporo Medical University School of Medicine, 291 Minami-1-jo Nishi 16-chome, Chuo-ku, Sapporo City, Hokkaido 060-8543, Japan

**Keywords:** CT, computed tomography, EGD, esophagogastroduodenoscopy, EGJ, esophagogastric junction, GERD, gastroesophageal reflux disease, Parahiatal hernia, Sliding hiatal hernia, Congenital defect, Mesh repair, Toupet fundoplication, Computed tomography

## Abstract

**Introduction:**

The parahiatal hernia is a rare type of diaphragmatic hernia in adults. Although there have been occasional reports of parahiatal hernias, few have reported simultaneous laparoscopic mesh repair of a parahiatal hernia with a hiatal hernia. This report describes laparoscopic mesh repair and fundoplication for a parahiatal hernia combined with an esophageal hiatal hernia.

**Presentation of case:**

A 39-year-old woman presented with left-side postprandial abdominal pain. Esophagogastroduodenoscopy revealed a parahiatal hernia and sliding hiatal hernia. Computed tomography (CT) showed that the stomach had prolapsed into the thorax from the outside of the left diaphragm. The preoperative diagnosis was parahiatal hernia with a hernial sac complicated by sliding hiatal hernia. Laparoscopic mesh repair was planned. The stomach had prolapsed on the left side of the esophagus and was extruded. The diagnosis of a parahiatal hernia with a hernial sac complicated by a sliding hiatal hernia was confirmed. The esophageal hiatal hernia was repaired using two non-absorbable sutures. The congenital defect was further reinforced with mesh fixed to the orifice of the adjacent parahiatal hernia. We performed Toupet fundoplication to treat gastroesophageal reflux disease and sutured the right diaphragmatic crus and stomach to prevent migration. The patient was discharged home on postoperative day 5.

**Conclusion:**

We encountered a patient with a parahiatal hernia complicated by a sliding hiatal hernia. The parahiatal hernia can be diagnosed by CT imaging. Preoperative diagnostic imaging can lead to appropriate treatment.

## Highlights

• The parahiatal hernia is a rare type of diaphragmatic hernia in adults
• This report describes laparoscopic mesh repair and fundoplication for a parahiatal hernia combined with an esophageal hiatal hernia.
• The parahiatal hernia can be diagnosed by CT imaging and preoperative diagnostic imaging can lead to appropriate treatment

## 1 Background

Parahiatal hernia is a rare type of diaphragmatic hernia in adults; its reported incidence ranges from 0.20 % to 0.35 % in patients undergoing surgery for hiatal hernia [1], [2]. This type of hernia is characterized by the presence of a diaphragmatic hernia defect immediately adjacent to an anatomically normal esophageal hiatus, unlike the Bochdalek hernia, which is located on the outer dorsal side of the diaphragm. Although there have been occasional case reports of parahiatal hernia, there are few describing simultaneous laparoscopic mesh repair of a parahiatal hernia with a hiatal hernia [1].

We describe a case of combined parahiatal hernia and esophageal hiatal hernia treated by laparoscopic mesh repair and fundoplication. The preoperative diagnosis and surgery details are discussed. This study has been reported in line with the SCARE 2020 Guideline [3].

## 2 Case presentation

**Fig. 1:**
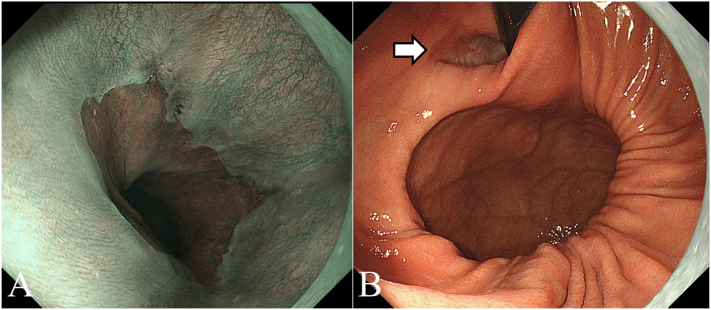
Esophagogastroduodenoscopy (EGD) findings. (A) Esophagogastric junction (EGJ) observed by narrow band imaging (NBI). The EGJ was enlarged and deemed to be grade A according to the Los Angeles classification. (B) Enlarged EGJ (arrow). The retroversion maneuver showed a saccular projection of the gastric lumen from the fornix.

**Fig. 2:**
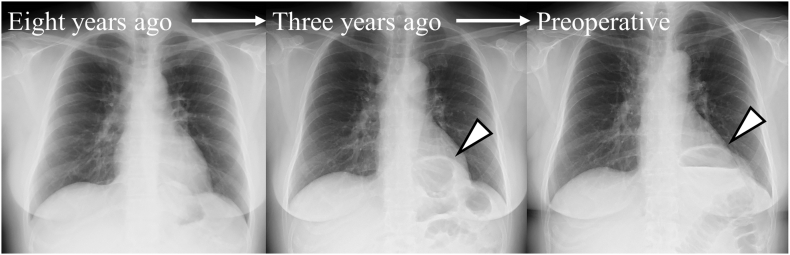
Radiographs of the chest showing increased abdominal gas in the thorax over time (arrowhead).

**Fig. 3:**
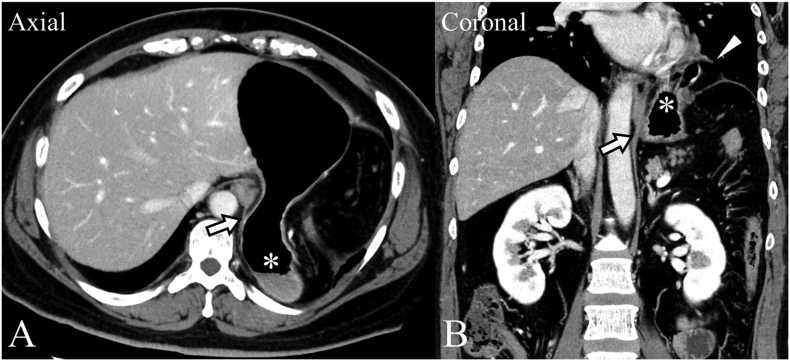
Preoperative contrast-enhanced computed tomography (CT). (A, B) Scan showing prolapse of the stomach into the thorax (*) from the outside of the left crus of the diaphragm (arrow). (B) A continuous membranous structure was observed from the diaphragmatic defect and covering the protruding part of the stomach (arrowhead).

The patient was a 39-year-old woman who presented with left-side abdominal pain after eating, which she had been aware of since 3 years of age. Her symptoms had worsened recently. She visited her local hospital and was referred to our department when a computed tomography (CT) scan indicated a possible esophageal hiatal hernia. The patient, who was 162 cm tall, weighed 75 kg, and had a body mass index of 28.6 kg/m^2^, had no history of smoking or upper abdominal surgery. Esophagogastroduodenoscopy (EGD) revealed an enlarged esophagogastric junction (EGJ), and narrow band imaging confirmed Los Angeles grade A gastroesophageal reflux disease (GERD) (Fig. 1A). During the retroversion maneuver, EGD showed a saccular projection in the gastric lumen from the fornix (Fig. 1B). A comparison of radiographic images of the chest, obtained before the onset of symptoms during late pregnancy and at the time of this presentation, showed an increase in the amount of abdominal gas in the thoracic cavity during the previous 3 years (Fig. 2). Enhanced CT imaging of the abdomen showed that the stomach had prolapsed into the thorax from the outside of the left crus of diaphragm (Fig. 3A, B). A continuous membranous structure from the diaphragmatic defect, covering the protruding part of the stomach, was observed and considered to be a hernial sac (Fig. 3B). We could not show the presence of a septum between the hiatus and the diaphragmatic defect. Thus, based on these findings, we diagnosed a parahiatal hernia with a hernial sac complicated by a sliding hiatal hernia, and performed laparoscopic hernia repair.

### 2.1 Surgical procedures

**Fig. 4:**
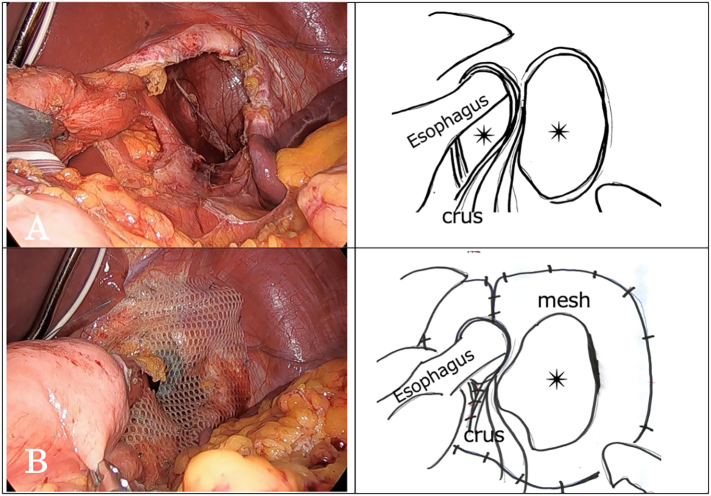
Intraoperative findings of the parahiatal hernia. (A) The stomach was ensnared in the parahiatal hernia defect. A continuous membranous structure was observed from the parahiatal hernia defect, which was considered to be a hernia. The sac and left lung were observed (asterisks). (B) The esophageal hiatus was mildly enlarged, and a hernial defect measuring 5 × 5 cm in diameter (asterisks) was found next to the left crus of the diaphragm. (C, D) Defect caused by the hiatal hernia and parahiatal (asterisks) hernia was repaired using a composite Symbotex™ mesh fixed using a titanium hernia stapler.

A small incision was made in the umbilical region, and surgery was initiated using a four-port approach. After the lateral hepatic segment was elevated, the gastric fundus was noted to be protruding through the hernial orifice. Return to the ventral side was straightforward using forceps traction. The content of the hernia was the stomach. The lesser omentum was incised, and the right crus of the diaphragm was exposed. Periesophageal dissection was advanced as far as possible to the ventral and dorsal sides. Next, the peritoneum around the hernial orifice was incised from the left side, and the left crus of the diaphragm was exposed. Dissection of the entire esophagus was completed and taped with a Penrose drain. The esophageal hiatus was mildly enlarged, and a hernial orifice measuring 5 × 5 cm in diameter was found next to the left crus of the diaphragm. The hernial sac had a peritoneal membrane. We diagnosed a parahiatal hernia with a hernial sac, complicated by a sliding hiatal hernia (Fig. 4A). The esophageal hiatus was repaired using two non-absorbable sutures. The orifice of the parahiatal hernia was too large to be closed with sutures. Because of the concern that the esophageal hiatus would open the adjacent parahiatal hernia, we decided to implant a mesh rather than suture the orifice. We performed a Toupet fundoplication for GERD and sutured the right diaphragmatic crus and stomach to prevent migration. A sheet of composite mesh (Symbotex™; Medtronic, Chicago, IL, USA) was trimmed to 10 × 10 cm, inserted in the abdominal cavity, and implanted at the site of the hernial orifice (Fig. 4B). The mesh was fixed using a Multifire Endo Hernia™ stapler (Medtronic). The surgical time was 232 min, with an estimated blood loss of 5 mL. No intraoperative blood transfusion was required.

### 2.2 Postoperative course

The postoperative course was uneventful, and the patient was discharged on postoperative day 5. After surgery, there was no symptom recurrence for 1 year.

## 3 Discussion

The diaphragm is derived from the septum transversum (anterior portion), pleuroperitoneal folds (posterolateral portion), and dorsal mesentery (posteromedial portion) [4]. A parahiatal hernia defect occurs toward the left side of the crus. Congenital parahiatal hernia is considered a posteromedial defect (because of failure of the dorsal mesentery to develop) within the diaphragm [4]. Owing to proximity, a parahiatal hernia is often confused with a hiatus hernia or Bochdalek hernia. The parahiatal hernia typically presents as epigastric discomfort, nausea, vomiting, and chest pain, particularly after meals, and early satiety. Characteristically, patients with a parahiatal hernia do not have symptoms of GERD unless it is complicated by a hiatal hernia. Therefore, because of the implications in terms of the choice of repair and overall outcome, it is important to identify these patients.

It is also important to differentiate a parahiatal hernia from a paraesophageal hiatal hernia to ensure appropriate treatment. The CT scan identified a hernial orifice adjacent to the left side of an esophageal hiatus, thus allowing for a preoperative diagnosis of parahiatal hernia in our case. Laparoscopic surgery offers a better field-of-view than open surgery; therefore, it is effective in terms of achieving an accurate intraoperative diagnosis. Although the parahiatal hernia is often diagnosed intraoperatively, we believe that a preoperative diagnosis would be more likely if surgeons were more aware of this entity.

Using “parahiatal” and “hernia” as search terms, we searched PubMed literature from 1968 to 2021, and we identified 20 cases of parahiatal hernia after the exclusion of irrelevant and secondary cases [1], [5], [6], [7], [8], [9], [10], [11], [12], [13], [14], [15]. Of these 20 cases, 9 were primary parahiatal hernia complicated by a sliding hiatal hernia (Table 1). The size of the hernial orifice was 4–5.5 cm. Laparoscopic mesh repair was selected for cases with fibrous thickening of the margins of the hernial orifice. Fundoplication was performed for all cases of hiatal hernia. The role of mesh repair in parahiatal hernia is still unclear because of the rarity of this entity and the lack of relevant literature. The reasons for using mesh to treat this case of parahiatal hernia were as follows: the patient had a hiatal hernia and an adjacent parahiatal hernia that enlarged further after the enlarged hiatal hernia was ligated with sutures; the parahiatal hernia was large, and closure with sutures would create high tension; and mesh can be used to repair a hiatal hernia. Surgery is recommended when symptoms are present, as in the present case, and for asymptomatic cases because of the risk of strangulation and gastric necrosis [16]. When a large defect prevents tension-free primary repair, the use of a composite mesh can achieve effective simultaneous repair of a parahiatal hernia complicated by a hiatal hernia. However, caution is necessary when placing the mesh to avoid aortic injury [17] and perforation of the EGJ [18] caused by mesh erosion.

**Table 1:** Overview of a primary parahiatal hernia complicated by a sliding hiatal hernia reported in the literature.

Case	Author	Year	Age	Sex	preoperative diagnosis	Operation	Size of hernia orifice (cm)	Fundoplication
1	Demmy et al. [5]	1998	64	F	Paraesophageal hernia	Laparoscopic suture repair	5	Nissen
2	Scheidler et al. [2]	2002	68	F	Paraesophageal hernia	Laparoscopic suture repair	–	Nissen
3			57	M	Paraesophageal hernia	Laparoscopic suture repair	–	Nissen
4	Palanivelu et al. [1]	2008	29	M	–	Laparoscopic mesh repair	5.5	Nissen
5			65	M	–	Laparoscopic mesh repair	4	Nissen
6	Ohtsuka et al. [9]	2013	51	F	Paraesophageal hernia	Laparoscopic mesh repair	–	180 anterior fundoplacation
7	Koh et al. [12]	2018	71	M	Paraesophageal hernia	Laparoscopic mesh repair	–	Partial posterior fundoplication
8	Staerkle et al. [13]	2019	60	F	(Parahiatal hernia)	Laparoscopic suture repair	4	Nissen
9	Our case	2021	39	F	Parahiatal hernia	Laparoscopic mesh repair	5	Toupet

The preoperative diagnosis of most cases was paraesophageal hernia, and all patients were diagnosed with a parahiatal hernia intraoperatively (Table 1). Among previously reported secondary parahiatal hernias, a case of postoperative parahiatal hernia was observed in a patient who had a hiatal hernia and underwent a fundoplication [1]. As a parahiatal hernia is considered to be caused by diaphragmatic dysplasia during embryonic period, it is highly likely that the hernia had already occurred at the time of the initial surgery, and that the diameter of the hernia portal was small and not confirmed intraoperatively. These cases required additional surgery because no preoperative diagnosis was made and no intraoperative diagnosis was made. Preoperative imaging and the consideration that there is a possibility of complications attributable to hiatal hernia and parahiatal hernia can lead to appropriate treatment, as in the present case.

## 4 Conclusions

We encountered a case of parahiatal hernia complicated by a sliding hiatal hernia. Parahiatal hernia can be diagnosed by CT imaging. Preoperative diagnostic imaging can lead to appropriate treatment.

## Consent

Written informed consent was obtained from the patient for publication of this case report and accompanying images. A copy of the written consent is available for review by the Editor-in-Chief of this journal on request.

## Availability of data and materials

The dataset supporting our conclusions is included within the article.

## Provenance and peer review

Not commissioned, externally peer-reviewed.

## Ethical approval

Not applicable.

## Funding

This research did not receive any specific grant from funding agencies in the public, commercial, or not-for-profit sectors.

## Guarantor

Ichiro Takemasa.

## Research registration number

This case report is not necessary because it is not First in Man study.

## CRediT authorship contribution statement

RM, TN, TI, TO, TK, and IT designed the research. RM, TN, and TK wrote the manuscript. All authors read and approved the final version of the manuscript.

## Declaration of competing interest

None.
